# Chemical cues and molecular mechanisms suspected in abiotic stress communication

**DOI:** 10.1242/jeb.251804

**Published:** 2026-02-11

**Authors:** Jiao Li, Lauric Feugere, Joerg Hardege, Sofia Vámos, Katharina C. Wollenberg Valero

**Affiliations:** ^1^ OdysysLab, University College Dublin, School of Biology and Environmental Science, Belfield Campus, Dublin 4, Ireland; ^2^ Marine Ecological and Evolutionary Physiology (MEEP) laboratory, Département de Biologie, Chimie et Géographie, Université du Québec à Rimouski, 300 Allée des Ursulines, Rimouski, QC G5L 3A1, Canada; ^3^ School of Natural Science, University of Hull, Cottingham Road, Kingston-upon-Hull HU67RX, UK; ^4^ Conway Institute, University College Dublin, Belfield Campus, Dublin 4, Ireland

**Keywords:** Transcriptomics, Metabolomics, Sensory biology, Chemical communication

## Abstract

For nearly a century, scientists have tried to resolve the sensory physiology of chemical communication caused by predation stress. Only recently have we evidenced that abiotic stressors from a changing world, such as heat and ocean acidification, also trigger chemical communication between aquatic organisms – which we dubbed abiotic stress communication. Generally, the behavioural and physiological response to stress-induced cues are well understood, whereas the molecular mechanisms – cue identities, pathways of release, and perception – of this stress communication remain unresolved. Here, we propose a framework to organize the existing evidence for candidate mechanisms involved in abiotic stress-induced chemical communication, focusing on heat and acidification as two major abiotic stressors with environmental relevance. Drawing on transcriptomic, metabolomic and behavioural evidence, we propose that stressor-specific communication likely involves multiple cues and parallel routes rather than a single mechanism, such as membrane-related processes. We call for integrative work that links -omics with chemical profiling and ecological function assays to uncover the mechanisms of abiotic stress communication.

## Teaser

**Summary:** Organisms facing climate stressors release chemicals informing others of risks. However, the sensory biology of this phenomenon remains unexplored. Here, we propose potential molecular mechanisms and the evidence uncovered for them.

## Introduction

Inter-individual communication is central to life, from bacteria (Taga and Bassler, 2003; Federle and Bassler, 2003) to plants (Gagliano et al., 2012) and animals (Laidre and Johnstone, 2013). One prominent form, chemical communication, involves the release of molecular cues that modify receiver behaviour or physiology (Regnier, 1971; Amo and Bonadonna, 2018; Sbarbati and Osculati, 2006). These cues mediate processes from predator avoidance to reproduction and social organization (Ohnstad et al., 2024; Roggatz et al., 2022; Fletcher et al., 2022; Kamio et al., 2022; Burks and Lodge, 2002). Chemical communication involves the production of a chemical cue, its release and transport into the environment, and its reception and integration by a receiver to trigger a response (Roggatz et al., 2022; Wyatt, 2014). Chemical communication occurs in all environments, but is especially important in freshwater and marine aquatic environments where the reliance on visual cues is limited (Hay, 2009; Roggatz et al., 2022).

Stress communication describes how stressed organisms release cues affecting others (Abreu et al., 2016; Barcellos et al., 2011; Feugere et al., 2021b). Most of the knowledge on stress chemical communication comes from research on alarm systems caused by biotic stressors, such as predation and injury (Crane et al., 2022). On land, insects emit alarm pheromones eliciting antipredator behaviours (Verheggen et al., 2010), and similar mechanisms occur in birds, rodents and humans (Brandl et al., 2022; Carnevali et al., 2020; Bombail, 2019; Bombail et al., 2018). Fish can recognize conspecifics by smell (Solomon, 1977, citing Wrede, 1932), and injured fish release alarm cues provoking defensive behaviours in receivers (Brown and Smith, 1997; Chivers and Smith, 1998). This was first described as Schreckstoff in minnows (von Frisch, 1938, 1942). Alarm systems have since been documented in arthropods (Verheggen et al., 2010), gastropods (Atema and Stenzler, 1977), echinoderms (Snyder and Snyder, 1970), amphibians (Hews, 1988) and fishes (Brown, 2003; Wisenden et al., 2004). Even non-injured animals emit disturbance cues upon being startled (Crane et al., 2022; Jordão and Volpato, 2000; Ferrari et al., 2010). Known alarm or disturbance cues include hypoxanthine-3-N-oxide (Brown et al., 2004), oxysterol sulphate and 5α-cyprinol sulphate (Li et al., 2024), and the glycosaminoglycans (GAGs) chondroitin sulphate (Mathuru et al., 2012) and keratan sulphate (Souza et al., 2007). Chemical communication is mostly studied in biotic stress contexts. Conversely, far less is known about whether abiotic stressors, such as warming and acidification, can likewise trigger inter-individual chemical stress communication. Despite decades of research since the first discoveries of biotic (von Frisch, 1938) stress chemical communication, the molecular identity and mechanisms of stress-induced chemical cue bouquets remain largely unknown (Stensmyr and Maderspacher, 2012). It also remains unclear whether abiotic and biotic stress cues and their pathways overlap. This represents a significant knowledge gap in the field of chemosensory biology and raises the important question of how animals might collectively respond to rapid global changes if chemical communication modulates species stress responses.

This knowledge gap, and two prior studies that observed behavioural changes in zebrafish and crayfish (Hazlett, 1985; Abreu et al., 2016), led us to the recent discovery that chemical communication extends to abiotic stressors. We showed that heat- or pH-stressed aquatic animals release cues that alter the growth, behaviour and gene expression of their receivers. This ripple effect propagates and amplifies stress responses within animal groups (Feugere et al., 2021a,b, 2023, 2025a). Prior to this, abiotic-related stress communication had only been anecdotally reported through behavioural studies in heat-stressed crayfish (Hazlett, 1985) and pH-stressed zebrafish (Abreu et al., 2016). In the context of rapid climate change, species will be increasingly exposed to abiotic stressors (Halpern et al., 2015; IPCC, 2019), especially higher temperatures owing to ocean warming and lower pH levels due to ocean acidification (Roggatz et al., 2022). Ocean acidification is caused by rising atmospheric CO_2_ released from human activities, which lowers seawater pH globally (Doney et al., 2009). Ocean acidification and ocean warming do not only manifest by average shifts, but also by increasing diel pH and temperature fluctuations (Bednaršek et al., 2022). Consequently, intense events such as short-term acidification, marine heatwaves and their co-occurrence (Landschützer et al., 2018; Burger et al., 2022) are becoming increasingly frequent and are superimposed on gradual global changes (Landschützer et al., 2018; Burger et al., 2022). These time scales matter for the individual organism, which will experience acute stress during its life course, and especially early development, potentially triggering stress communication.

The developmental stage of organisms may fundamentally shape these pathways. Early life stages are capable of chemical communication (Lucon-Xiccato et al., 2020; Atherton and McCormick, 2015; Cao and Li, 2020). Embryos lacking a differentiated epidermis are more permeable, and may release chemical cues (but also uptake them) through loosely organized outer cell layers (Le Guellec et al., 2004). Moreover, the presence of a chorion limits the permeability for certain size classes of molecules (Pelka et al., 2017). Once the functional epidermis is developed, permeability to outgoing signals may be more limited or utilize other pathways, such as urine excretion.

**Fig. 1.**
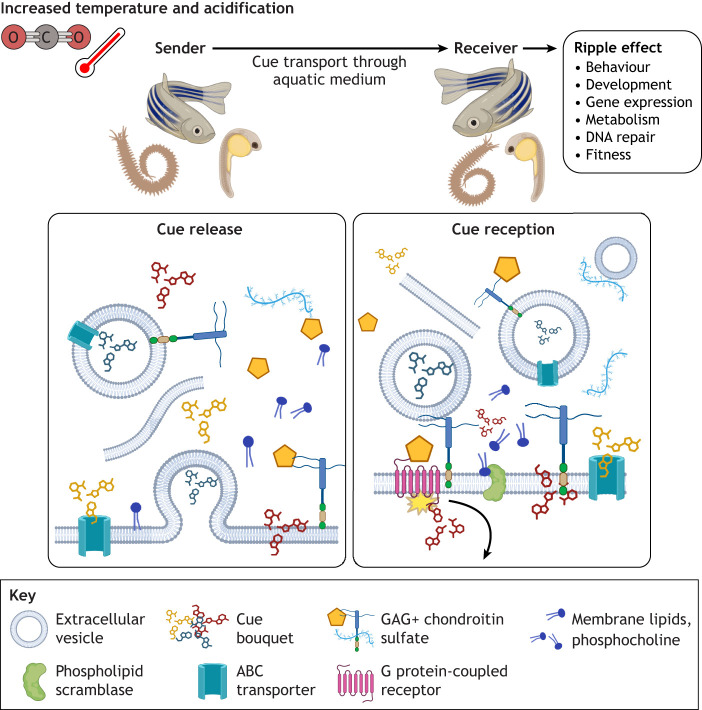
**Schematic overview of the main molecular mechanism of abiotic stress communication.** Upon exposure to heat and acidification, pathways are activated in organisms that initiate the release of stress cues into the environment by senders. These are then perceived by receiver organisms, in which specific molecular pathways are triggered which alter properties of the receiver. This ripple effect propagates and amplifies stress responses between individuals. Created in BioRender. Wollenberg Valero, K. (2025) https://BioRender.com/g635dq1. This figure was sublicensed under CC-BY 4.0 terms.

In this Commentary, we discuss the evidence of abiotic stress communication from our previous studies on zebrafish embryos (*Danio rerio*), sea bream (*Sparus aurata*) and marine ragworms (*Hediste diversicolor*; Feugere et al., 2021b, 2023; Beine et al., 2024) exposed to elevated temperature and acidified pH. We discuss possible molecular mechanisms and propose an evolutionary raison d’être for abiotic stress communication and its implication in a fast-changing world (Fig. 1). We have identified five mechanisms with evidence of involvement in abiotic stress chemical communication: (1) lipids and membranes, (2) proteins, (3) extracellular matrix (ECM), (4) other small molecules and (5) immune system entanglement (Table 1). We present these mechanisms in the next sections.

**Table 1.** Five proposed molecular mechanisms of abiotic stress chemical communication

Molecular pathways	Hypotheses and evidence for their involvement in abiotic stress chemical communication
**Lipids and membranes**	
Release	Hypothesis: abiotic stressors remodel membranes, releasing lipids or membrane fragments. Evidence: *Hydra* uses extracellular vesicles (ECVs) for social signalling through the medium.
Cues	Hypothesis: abiotic stress cues are lipids released under abiotic stress. Evidence: lipids are known cues in chemical communication. Medium from heat-stressed zebrafish embryos contains phosphocholines and phosphatidylcholines.
Reception	Hypothesis: membrane-bound lipids sense stress cues. Evidence: receivers of heat-stress cues in zebrafish upregulate *prg4a* (linked to phosphocholine) and the scramblase *xkr8.2* (which flips membrane phospholipids). *xkr8.2* is upregulated in lateral line hair cells, which may detect chemical cues. Ragworms receiving cues from pH-stressed fish differentially express *gpr45*, a lipid-signaling G-protein coupled receptor (GPCR).
**Proteins**	
Release	Hypothesis: proteins such as ABC transporters secrete abiotic stress cues. Evidence: proteins including ABC transporters export signalling cues across kingdoms and are involved in the cellular stress response. No evidence yet for their role in abiotic stressor cue release.
Cues	No evidence for the involvement of proteins/peptides in abiotic stress signalling.
Reception	Hypothesis: chemosensory, transport and receptor proteins are involved in abiotic stress cue reception. Evidence: zebrafish embryos exposed to heat stress and to heat-stressed conspecific cues upregulated chemosensory receptor genes (*ora3*, *olfcb1*). Marine ragworms exposed to pH-stressed fish cues modulated transcription of several receptors (*trpc6*, *otop*, otog, *gbra6* and several GPCRs incl. *gpr45*, *fmar*), transport (*slc4a10*) and ABC transporter (*abcb6*) proteins.
**Extracellular matrix (ECM)**	
Release	Hypothesis: ECM secretes abiotic stress cues. Evidence: heat stress alters the expression of ECM-related genes linked to glycosaminoglycans (GAGs). GAGs are alarm cues in fish.
Cues	Hypothesis: abiotic stress cues are ECM components. Evidence: ECM components such as hypoxanthine-3-N-sulphate and chondroitin sulphate are alarm cues. There is no evidence for their roles in abiotic stress signalling, suggesting that biotic and abiotic stress communication may differ.
Reception	Hypothesis: ECM detects abiotic stress cues. Evidence: zebrafish embryos exposed to abiotic stress cues upregulated an ECM adaptor linking proteoglycans (*matrilin 3a*), and genes linked to GAG metabolism (*krt17, krt97, chs1, chst2b* and *prg4a*).
**Other small molecules**	
Release	Hypothesis: small molecules pass through membranes and lead to abiotic stress cues. Evidence: heat-stressed zebrafish embryos activate small molecule metabolism including pentose phosphate (PPP) and methionine metabolism pathways (involving *gpia* and *pgm1*).
Cues	Hypothesis: abiotic stress cues are small molecules. Evidence: the medium from heat-stressed zebrafish embryos contain primary metabolites, including carbohydrates (PPP), sulphur-containing compounds (transsulphuration pathway; e.g. 3-mercaptolactate).
Reception	No evidence for the role of specific small molecules in receiving abiotic stress cues.
**Immune system**	
Release	No evidence for immune system involvement in abiotic stress cue release.
Cues	Hypothesis: abiotic stress cues stem from immune responses. Evidence: damage-associated molecular patterns (DAMPs; e.g. oxidised lipids or degraded ECM fragments) can mimic pathogen-associated molecular patterns (PAMPs) and activate innate immunity via epithelial detection and inflammatory signaling. DAMPs are often purine derivatives. The alarm cue hypoxanthine (Schreckstoff), a purine derivative, may activate immune responses in receivers.
Reception	Hypothesis: abiotic stress cues induce immune pathways. Evidence: zebrafish embryos exposed to heat stress cues downregulated genes involved in immunity signalling (*si:ch211-214b16.2* and *il-1β*).

We propose the mechanisms underlying release of the abiotic stress cues by the sender, their molecular identity and, lastly, reception by the receiver. Detailed references for each point are given in the main text.

## Lipids and membranes

### Temperature-induced membrane remodelling as possible lipid cue release mechanism

Membrane lipid composition varies in response to environmental stressors through both homeoviscous and homeophasic adaptation maintaining their fluidity and phase stability (Jin et al., 2021; Sinensky, 1974; Vigh et al., 1998; Hazel, 1995, 1997). Warming lowers acyl chain unsaturation (Holm et al., 2022), and can cause shifts in lipid phase behaviour, including transitions to non-lamellar phases such as the inverse hexagonal phase. Heat stress can also alter headgroup composition and cholesterol content (Vigh et al., 1998; Hazel, 1997). For example, in *Tetrahymena*, heat increases membrane phosphatidylcholines (Nozawa, 2011), a lipid class derived from phosphocholine (Li and Vance, 2008). Likewise, experimental ocean warming and acidification treatments altered the muscle membrane lipidome of *Pandalus borealis* shrimp (Feugere et al., 2025b).

Therefore, we propose that lipid monomers deriving from stress-induced membrane remodelling could act as abiotic stress cues. However, their amphiphilic nature limits their persistence in water, and they tend to form micelles or surface monolayers (Pichot et al., 2013). A different mechanism of transport in aquatic environments would require monomers to be stabilized by carrier proteins (Mangiacotti et al., 2023), or to self-assemble into micelles or vesicles (spheres of lipid bilayers, extracellular vesicles or ECVs). ECVs may carry and release other chemical cues as cargo into the environment. The density of nano-sized ECVs is around 1.10–1.19 g ml^−1^ (van der Vlist et al., 2012), compared with 1.03 g ml^−1^ for seawater (depending on temperature and/or salinity; McDougall and Parker, 2011) and 1 g ml^−1^ for freshwater. Therefore, ECVs should remain suspended in seawater but slightly sink in freshwater, making them likely candidates to transport signalling molecule cargo between organisms in both media. In fact, the aquatic invertebrate *Hydra vulgaris* has been shown to release ECVs containing molecular messages for other individuals into its culture medium (Moros et al., 2021).

### Lipids and derivatives as abiotic stress cues

Zebrafish embryos kept for 24 h post-fertilization (hpf) under a fluctuating heat stress activated two key pathways (glycerophospholipid and ether lipid metabolism) and released lipids into the medium. These included fatty acyls, glycerolipids, sphingolipids, glycerophospholipids and steroid esters (Feugere et al., 2023), which links membrane lipid turnover to heat stress signalling. Among these lipids, phosphocholine and glycerophosphocholine (a derivative of phosphocholine) stood out. Both of these lipids were previously shown to be upregulated in heat-stressed fish tissues (Karakach et al., 2009; Zhang et al., 2022). Lipids, such as sphingolipids, are already known for protecting cells under heat stress and acting as molecular messengers (Balogh et al., 2013; Török et al., 2014), mediating mating in reptiles (Goldberg et al., 2017), guiding larval migration in lampreys (Li et al., 2018), communicating the presence of copepod predators to dinoflagellate prey (Selander et al., 2015), and marking group identity in catfish (Matsumura et al., 2007). Furthermore, lipids can serve as coral settlement cues (Jorissen et al., 2021). In zebrafish, the yolk sac likely serves as an abundant source of such lipids (Miyares et al., 2014). Moreover, crustacean pheromones and contact cues may be simple, heat-stable lipids (Takahashi, 1974; Zhang et al., 2011). Altogether, there is strong evidence that abiotic stress can turn membrane lipids into messages.

### Uptake of lipids from abiotic stress medium

Receptors for free phospholipids in aquatic environments are largely unknown. Although lipid head group exposure can trigger cell-to-cell signals (e.g. in apoptosis), this mechanism is understood intracellularly (Fadok et al., 1998), but under-researched in inter-organismal communication. However, lipid signals can integrate into cell membranes through membrane fusion and alter fluidity, curvature or receptor distribution, making membranes themselves interpreters of environmental cues (Vigh et al., 1998). Contact with ECVs could bulk-transfer metabolic and stress-related information. In zebrafish, sensory cells such as those in the lateral line might detect environmental changes via lipid uptake. For instance, 24-hpf zebrafish embryos exposed to thermal stress cues upregulated genes involved in phospholipid and anion transport. This included the scramblase *xkr8.2*, which flips membrane phospholipids and is both highly expressed in lateral line hair cells and upregulated by stress cues (Feugere et al., 2023; Elkon et al., 2015). Moreover, multiomics analyses functionally linked a gene associated with the ECM (see below), *prg4a*, to phosphocholine, and its consistent upregulation across several independent experiments strengthens its candidate role in cue response (Feugere et al., 2023). In marine ragworms grown in a future ocean regime (adjusted diel and seasonal temperature and pH cycles), *gpr45*, a lysophosphatidic acid-like lipid-signalling G-protein coupled receptor (GPCR), is differentially expressed in response to exposure to the metabolites of *Sparus aurata* sea bream exposed for an hour to acidified pH (Marchese et al., 1999; Cui et al., 2016; Geraldo et al., 2021; Kiss et al., 2012; Lee et al., 2023; Beine et al., 2024). This shows that lipid signals present in abiotic stress media can be taken up by receivers, again supporting their role in abiotic stress communication.

## Proteins

### Protein-mediated abiotic stress cue release

Although there is no evidence for secretory proteins actively transporting abiotic signalling cues into the aqueous environment, some protein candidates warrant further consideration. For example, ATP binding cassette (ABC) transporters are a ubiquitous large family of proteins that use ATP to actively transport molecules across membranes (Theodoulou and Kerr, 2015), including ions, organic molecules of different polarity, lipids, peptides and small proteins. Beyond individual molecule export, some ABC subfamilies are also implicated in vesicle formation or cargo loading, suggesting they might facilitate cue packaging into lipid vesicles or microdomains (Theodoulou and Kerr, 2015). ABC transporters are known to export signalling cues across kingdoms. In tunicates, they guide sperm cell motility by exporting signalling lipids (Kassmer et al., 2020). In plants, they mediate phytohormone transport within tissues and into the soil (Borghi et al., 2015; Sasse et al., 2015), support chemical kin recognition (Biedrzycki et al., 2011; Bailly, 2014) and facilitate the release of small volatile organic compounds (Adebesin et al., 2017). ABC transporters also contribute to the heat stress response. In yeast, heat-induced ABC transporters deliver ergosterol to plasma membranes, increasing their fluidity and integrity (Godinho et al., 2021). In pigeon pea, ABC genes are directed by stress-responsive promoters, to export bioactive compounds such as hormones and signalling molecules (Niu et al., 2021).

### Protein-mediated stress cue uptake

GPCRs, vanilloid receptors [transient receptor potential (TRP) channels], scavenger receptors and GABA receptors mediate pheromone and kairomone detection (Fortes-Marco et al., 2013), environmental sensing and systemic physiology regulation, making them strong candidates for stress cue detection. In adult fish, the olfactory bulb processes chemical cues (Hamdani and Døving, 2007), but this system is still maturing in embryos and larvae (Li et al., 2005). Zebrafish express three main olfactory GPCRs: olfactory receptors (Liberles and Buck, 2006; Hashiguchi and Nishida, 2007; Liberles, 2009), trace amine-associated receptors, vomeronasal receptors (V1Rs, V2Rs), plus other pheromone-detecting GPCRs (Pfister and Rodriguez, 2005). Cues binding to GPCRs activate G-protein pathways, and open ion channels via increased cAMP (Kandel et al., 2000). The lateral line system expresses TRP and purinergic chemoreceptors (Corey et al., 2004; Xiang et al., 1999; Quick et al., 2012; Muñoz et al., 1999), and also detects chemical cues via mechanosensory hair cells (Coombs et al., 2013; Dijkgraaf, 1963; Webb, 2013). Recently, 45 novel chemoreceptors were discovered in zebrafish hair cells, including 19 olfactory GPCRs, TRPs and pheromone receptors (Desban et al., 2022). We also found that zebrafish embryos exposed to heat and heat-induced stress cues simultaneously, but not to heat alone, altered the expression of the chemosensory receptor genes *ora3* and *olfcb1* (Feugere et al., 2023). In marine ragworms, pH-stress-exposed fish cues upregulated TRPC6, downregulated OTOP and OTOG, and modulated GBRA6 and several GPCRs such as GPR45 and FMAR (Beine et al., 2024; Berna-Erro et al., 2014). These patterns suggest responses to stress cues induced by altered extracellular pH or heat stress, either directly or indirectly. In marine ragworms, exposure to stress cues from pH-stressed sea bream induced distinct gene expression patterns (Beine et al., 2024). For instance, stress cues upregulated *slc4a10* whereas direct pH stress downregulated it. Notably, stress cues downregulated *abcb6* (Ye et al., 2020; Rakvács et al., 2019), an ABC transporter with membrane phospholipid binding function (Lee et al., 2023). Owing to their secretory nature, ABC transporters are unlikely to be receptors for abiotic stress cues, but they could function in intracellular reprogramming following the detection of stress cues. However, ABC transporters can internalize quorum sensing signals in microorganisms (Taga and Bassler, 2003), or even ‘steal’ xenosiderophores, which are iron-binding molecules secreted by other species (Liu and Kakeya, 2020). Besides their signalling role being mostly known from plants and microbes, the upregulation of ABC transporters in heat-stressed aquatic organisms may simply reflect a general cellular protection response (Lauritano and Ianora, 2020).

## Extracellular matrix

### Damage-induced release of stress cues

Alarm cues are associated with injury. Schreckstoff was first identified in skin extract triggering startle responses in conspecifics (Smith, 1979). von Frisch (1942) noted that Schreckstoff was only produced by epidermal cells. In fish, the ECM is essential for epithelial integrity and supports the mucus layer enriched in GAGs (Syed et al., 2022; Frantz et al., 2010). This supports the idea that membrane damage caused by abiotic stress could cause the passive leakage of cytoplasmic components or unstructured membrane fragments, including parts of the ECM. This structure is tightly linked to cell membranes and includes proteoglycans known to carry potential cue components, including GAGs. Known ECM alarm cues include GAGs such as chondroitin sulphate and mucin proteins (Mathuru et al., 2012). The original Schreckstoff, hypoxanthine-3-N-oxide (Parra et al., 2009), is a chemical relative of hypoxanthine and a derivative of adenine. The latter is part of the ECM structure in zebrafish (Deis et al., 2025). Altogether, this supports the hypothesis that abiotic stressors lead to the release of GAGs via disrupted membrane fragments with the ECM attached. However, there was no evidence for specific ECM fragments involved in abiotic stress communication in our prior study in zebrafish (Feugere et al., 2023).

### Extracellular matrix as abiotic stress cue receiver?

The ECM is not purely structural. It also modulates signalling through ligand binding and receptor presentation (Kim et al., 2011), making it a likely interface for receiving and releasing stress cues. In 24-hpf zebrafish embryos exposed to medium from heat-stressed conspecifics, we showed that heat stress and stress cues alter the expression of ECM-related genes, such as matrilin 3a (*matn3a*, an ECM adaptor linking proteoglycans; Feugere et al., 2023; Paulsson and Wagener, 2018), proteoglycan 4a (*prg4a*), and genes related to the GAGs keratan sulphate and chondroitin sulphate biosynthesis (*chs1*, *chst2b*, *krt17*, *krt97*) (Feugere et al., 2023). This suggests a role of ECM molecules in abiotic stress-mediated cue reception. Multiomics linked *prg4a* to phosphocholine, a lipid that is a potential heat stress cue, and was consistently upregulated across our experiments. In marine ragworm, receivers of stress cues from pH-stressed sea bream altered expression of four ECM-related genes (*ust*, *xylt2*, *b4galt7*, *b3galt4*), involved in chondroitin sulphate metabolism (Beine et al., 2024). Altogether, this supports the role of ECM in cue perception within and across different species.

## A potential role for other small molecules

In addition, carbohydrates (and conjugates) were identified in the medium of heat-stressed zebrafish. These cues, such as deoxyribose, likely result from heat-induced metabolic shifts, including glycolysis and the key redox regulator pentose phosphate pathway (PPP). Genes associated with PPP (*gpia* and *pgm1*) were upregulated in the heat-stressed zebrafish embryos (Feugere et al., 2023; Stincone et al., 2015; Karakach et al., 2009), which links a heat-induced metabolic process to its products identified in the stress medium. In fact, carbohydrates are known information carriers (Gabius et al., 2004). Likewise, sulphur-containing compounds are known alarm cues (Brechbühl et al., 2013; McLean et al., 2021; Miyazaki et al., 2006; Li et al., 2024) and two that are involved in methionine metabolism (Sörbo, 1987; Andreeßen et al., 2018) were identified in the thermal stress medium of zebrafish embryos (as 3-mercaptolactate and 2-oxo-4-methylthiobutanate; Feugere et al., 2023). 3-Mercaptolactate is synthesized by lactate dehydrogenase A (*ldha*), which we showed to be heat-induced (Feugere et al., 2023). 3-Mercaptolactate is excreted in urine and induces anxiety-like behaviours in mice (Nagahara et al., 2013; Akahoshi et al., 2020; Andreeßen et al., 2018; Song et al., 2019).

The question that follows is then, how can these small molecules be released from the thermally stressed individual? Metabolites, if they are hydrophobic, or polar and small, may readily diffuse through the cell membrane passively (Cooper, 2000). Two examples for passive diffusion of signalling molecules are the alkaloid neurotoxins saxitoxin and tetrodotoxin. These molecules evolved as chemical defences, but also function as inter-organismal cues (Roggatz et al., 2019). Their ability to passively cross membranes allows them to act without specialized release pathways (Tikhonov and Zhorov, 2018; Van Theemsche et al., 2020). Similar to this model, smaller, more volatile metabolites are strong candidates for cues that can be detected at a distance from their source (Alberts and Werner, 1993; Konishi, 1970; Pozzer et al., 2022). In our previous experiment, classical alarm cues, including hypoxanthine-3-N-oxide, trigonelline and homarine (Poulin et al., 2018; Parra et al., 2009), were released by control zebrafish embryos rather than the heat-stressed ones (Feugere et al., 2023), suggesting chemical differences between abiotic stress and alarm cue bouquets. In summary, although the exact mechanism of small molecule cue release is unknown, and no evidence exists yet for the role of small molecules in abiotic stress communication, we showed that some are released into the medium by heat-stressed developing embryos as metabolic products of the primary metabolism.

## Immune system entanglement

The immune system may misinterpret abiotic stress cues as pathogen-associated molecular patterns (PAMPs), triggering responses through shared pathways. Damage-associated molecular patterns (DAMPs), such as oxidized lipids or degraded ECM fragments, can mimic PAMPs, activating innate immunity via epithelial detection and inflammatory signalling (Tang et al., 2012; Varki, 2011). DAMPs are often purine derivatives (Russo and McGavern, 2015), such as hypoxanthine (Schreckstoff). In threespine stickleback, predator and alarm cues similarly modulated immune and signalling pathways (Sanogo et al., 2011). In zebrafish embryos, exposure to heat and heat stress cues downregulated *si:ch211-214b16.2*, likely a NOD2 ortholog (Feugere et al., 2023), and heat stress cues downregulated *il-1β* involved in immune pathways and inflammation, possibly indicating immune suppression (Feugere et al., 2021b). Although NOD2 typically detects bacterial peptidoglycans, its modulation here may reflect recognition of stress-modified glycosaminoglycans in ECM-bound cues. Notably, *tlr18*, a fish-specific toll-like receptor associated with lipid recognition and expressed in lateral line hair cells, was activated by stress cues of heat-exposed zebrafish embryos (Nie et al., 2018; Elkon et al., 2015; Feugere et al., 2023). Toll-like receptors might therefore recognize stress-induced lipids or ECM fragments, triggering MAPK or NF-κB cascades and fear-like behaviours (Kirsten et al., 2018). Overall, this indicates that the reception of thermal stress cues modulates the immune system of receivers.

## Why would abiotic stress communication exist?

In this Commentary, we describe the phenomenon of abiotic stress communication and discuss possible underlying molecular mechanisms. We have shown abiotic stress induces chemical communication, defined as information exchange, irrespective of intentionality, by means of chemical cues affecting their receivers (Wyatt, 2014). Zebrafish embryos receiving heat stress cues exhibited stress-like molecular responses, developed faster and were hypoactive, similar to heat-stressed conspecifics. Marine ragworms changed their burrowing, freezing and escaping behaviour when receiving cues from pH-stressed conspecifics as well as pH-stressed sea bream (Feugere et al., 2021a; Beine et al., 2024). The purpose of injury-induced biotic stress communication is likely for receivers to perceive the predation risk, without intentionality by the sender (Crane et al., 2022; Chivers et al., 2012). This would make them ‘honest’ biochemical byproducts of stress that unintentionally inform others (Darwin, 1871; Andersson, 1994; de Groot and Smeets, 2017). Stress communication, whether biotic or abiotic, may in fact be widespread across social animal species owing to the evolutionary conservation of stress response mechanisms (Brandl et al., 2022). The evolutionary basis of abiotic stress communication is less obvious than for alarm signals caused by predation, where the threat is immediate and acute.

For abiotic stress communication, all mechanisms mentioned above similarly point at an unintentional and involuntary release of stress cues into the environment, originating from cell damage or changes in the primary metabolism of the sender. Therefore, we discuss four possible roles for abiotic stress communication, which are to: (i) be informed about others' physiological status, (ii) coordinate stress responses in a group and (iii) mitigate abiotic stress effects through either priming for the abiotic stressor or initiating stress responses, for instance by completing embryonic development faster or displaying specific behaviours (escaping, freezing, moving to another habitat). Lastly, (iv) abiotic stress cues may overlap with biotic stress cues and therefore cause similar reactions in receivers.

### Role 1: physiological status

Akin to social stress transmission, abiotic stress communication may help to inform others of stress (Brandl et al., 2022). One may argue that there is no need to have a chemical stress communication system as the individual perceives the abiotic stress itself. Animals are known to gather as much information as possible, including socially acquired information, even if inadvertently (Dall et al., 2005). Species are also known to eavesdrop on alarm and disturbance cues of other species (Turner et al., 2023). Therefore, the ability for an animal to detect another animal’s stress cues can inform it about its health status or whether a habitat is suitable.

### Role 2: coordination of stress group responses

An abiotic stress communication system may be particularly useful to coordinate responses in social species (Grunst and Grunst, 2024). In a mechanism akin to quorum sensing (Li and Nair, 2012), a minimum threshold of stress cues may help the group coordinate its stress response to the threat, acting as honest social signals favouring group cohesion (Dall et al., 2005). This aligns with alarm and disturbance cue studies showing group responses depending on the stress experience of senders and receivers, with dose- and threshold-dependent patterns (Goldman et al., 2019, 2020). Likewise, as stress responses and stress perception vary among individuals (Guscelli et al., 2019; Melanson et al., 2023), abiotic stress communication may serve as a social coordination checkpoint that matches the group response to the level of the abiotic stressor, to coordinate responses such as developmental speed. This social buffering may counterbalance stress transmission to prevent maladaptive costs when environmental changes occur on longer time scales (discussed in Gilmour and Bard, 2022).

### Role 3: mitigation of abiotic stress effects

Abiotic stress communication may be a hormetic mechanism, a low-intensity stressor priming the stress response and reducing the damage caused by additional abiotic stressors, such as improving DNA repair (Feugere et al., 2025a). Stress cues released by heat-stressed individuals accelerated growth in naive receivers and amplified it in heat-stress-exposed receivers (Feugere et al., 2021a,b, 2023). Abiotic stress communication may accelerate individual growth within cohorts to increase survival, as developed animals are more equipped to cope with the stress than developing ones (Hamdoun and Epel, 2007; Cowan et al., 2024). Abiotic stress communication may prime animals through the activation of the molecular pathways, including the immune system defenses, needed to respond to the abiotic stressor.

### Role 4: overlap with biotic stress cues

In our Commentary, we summarized multiple lines of evidence for membrane lipids and/or membrane fragments including the ECM being involved in abiotic stress communication. Similarly, lipids have multiple functions in social signalling, and the known injury-induced Schreckstoff components are most likely components of the ECM. Therefore, if abiotic stressor-induced membrane fragments come into contact with another organism, they may interpret the cue as alarm signals, as the same compounds are carried via the ECM or membrane lipids.

## Future research directions

Abiotic stress communication appears across diverse organisms, tissues and developmental stages. Given that environments are increasingly challenging to organisms, it becomes crucial to further our understanding of its molecular mechanisms if abiotic stress communication has the potential to amplify stress responses. In zebrafish, early-life responses to stress cues may involve olfactory pathways, but confirmation in adults is still needed (Feugere et al., 2023). Mechanisms likely vary across taxa and stressors, e.g. heat, low pH and osmotic stress, yet converging patterns emerge. Chondroitin sulphate and other glycosaminoglycans recur across species, and many abiotic stressors affect membrane structure, implicating conserved pathways such as vesicle-mediated transport and ECM remodelling in cue propagation. Unlike alarm cues such as Schreckstoff, which are often species-specific (von Frisch, 1942), abiotic stress communication may rely on broadly shared components. This aligns with the concept of a cue bouquet composed of diverse molecules, including lipids, proteins, sugars or ECM fragments, acting in concert. In terrestrial species, lipids often require protein binding for signalling (Mangiacotti et al., 2023; Telser, 2002) and similar packaging into lipid vesicles enriched with small molecules and glycoproteins may analogously occur in aquatic systems. However, decades of research on Schreckstoff (e.g. Mathuru et al., 2012) have evidenced that it is extremely difficult to dissect the role of cues in signalling bouquets, calling for future research to identify what chemicals among stress metabolites act as effective stress cues driving abiotic stress communication. Acknowledging these limitations, we call for discussions among molecular biologists, ecophysiologists, chemical ecologists and evolutionary scientists to resolve this outstanding question. A unified framework is needed to recognize chemical stress communication as a widespread, fundamental process. Uncovering more about this mechanism will require systemic, multiomic and behavioural approaches (Hebets et al., 2016; Brunetti et al., 2018).
